# Digital Biometry as an Obesity Diagnosis Tool: A Review of Current Applications and Future Directions

**DOI:** 10.3390/life14080947

**Published:** 2024-07-28

**Authors:** Florence Porterfield, Vladyslav Shapoval, Jérémie Langlet, Hanen Samouda, Fatima Cody Stanford

**Affiliations:** 1 Department of Medicine-Metabolism Unit, Massachusetts General Hospital, Boston, MA 02114, USA; fstanford@mgh.harvard.edu; 2 Clinical Pharmacy and Pharmacoepidemiology Research Group, Louvain Drug Research Institute (LDRI), Université Catholique de Louvain—UCLouvain, 1200 Brussels, Belgium; 3 Business Development Office, Luxembourg Institute of Health, 1445 Strassen, Luxembourg; 4 Nutrition and Health Research Group, Department of Precision Health, Luxembourg Institute of Health, 1445 Strassen, Luxembourg; hanene.samouda@gmail.com; 5 Department of Medicine-Neuroendocrine Unit and Department of Pediatrics-Endocrinology, Massachusetts General Hospital, Harvard Medical School, Boston, MA 02114, USA

**Keywords:** digital biometry, obesity, body composition, 3D body scanners, 2D body scanners

## Abstract

Obesity is a chronic relapsing disease and a major public health concern due to its high prevalence and associated complications. Paradoxically, several studies have found that obesity might positively impact the prognosis of patients with certain existing chronic diseases, while some individuals with normal BMI may develop obesity-related complications. This phenomenon might be explained by differences in body composition, such as visceral adipose tissue (VAT), total body fat (TBF), and fat-free mass (FFM). Indirect measures of body composition such as body circumferences, skinfold thicknesses, and bioelectrical impedance analysis (BIA) devices are useful clinically and in epidemiological studies but are often difficult to perform, time-consuming, or inaccurate. Biomedical imaging methods, i.e., computerized tomography scanners (CT scan), dual-energy X-ray absorptiometry (DEXA), and magnetic resonance imaging (MRI), provide accurate assessments but are expensive and not readily available. Recent advancements in 3D optical image technology offer an innovative way to assess body circumferences and body composition, though most machines are costly and not widely available. Two-dimensional optical image technology might offer an interesting alternative, but its accuracy needs validation. This review aims to evaluate the efficacy of 2D and 3D automated body scan devices in assessing body circumferences and body composition.

## 1. Introduction

Obesity is a chronic multifactorial disease and a significant public health concern due to its high prevalence worldwide and the possible associated health issues, including cardiometabolic, vascular, kidney, and pulmonary diseases as well as some cancers and neurodegenerative disorders [1,2,3]. Paradoxically, research has shown that obesity might favorably affect the prognosis of patients with existing cardiovascular, renal, and respiratory diseases as well as some cancers. There are also individuals with “normal weight” as assessed by body mass index (BMI) that might develop complications that are traditionally thought to be associated with obesity [4,5,6,7,8,9,10,11].

Obesity is largely defined using BMI given the ease of use, accessibility, and low cost of this diagnostic method. However, using BMI alone confers significant limitations in obesity diagnosis. Beyond BMI, specific aspects of body composition—specifically, increased intra-abdominal adiposity or visceral obesity, rather than the sole gross corpulence as assessed by BMI—appear to increase the risk of obesity-associated diseases. In particular, sarcopenic obesity, which is characterized by decreased muscle mass, fat infiltration in the skeletal muscle, and increased total and regional fat mass, contributes to inflammation, insulin resistance, and several other obesity-related complications [12,13]. In contrast, subcutaneous adiposity has shown a protective effect [14]. These different phenotypes of obesity due to body composition dysregulations have been suggested to explain the obesity paradox, or why patients with normal weight as defined by BMI have metabolically unhealthy (MU) phenotypes whereas patients with obesity have metabolically healthy (MH) phenotypes [15,16].

## 2. Indirect Estimation of Body Composition

**Figure 1:**
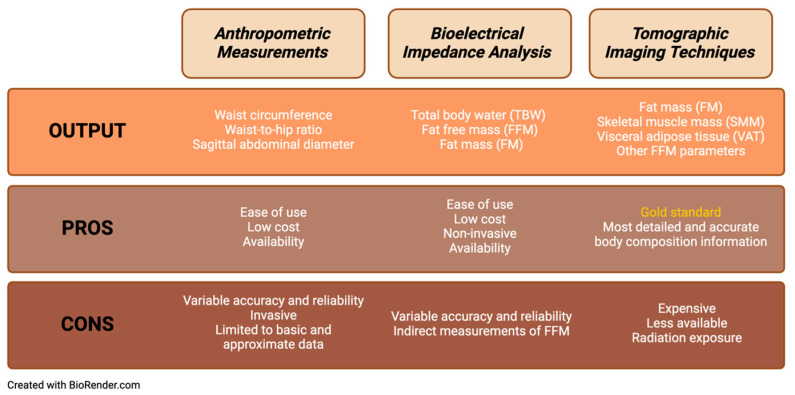
Comparison of methods for assessing body composition.

Indirect measures of obesity such as body circumferences, skinfold thicknesses, and bioelectrical impedance analysis (BIA) devices are helpful clinically and in epidemiological studies as they can be applied to many patients. They can also be used to predict body compartment volumes and their relationship with several health issues. The various methods of measuring body composition are outlined in Figure 1.

### 2.1. Anthropometric Measurements

Several anthropometric measurements have been proposed to assess visceral adiposity, such as waist circumference, waist-to-hip ratio, and sagittal abdominal diameter [17,18]. Although easy to measure and inexpensive, these markers cannot distinguish between visceral adipose tissue (VAT) and subcutaneous abdominal adipose tissue (SAAT), thus limiting the accurate estimation of VAT [19]. Other body circumferences and skinfold thicknesses have been used to assess total body fat and muscle mass [20,21]. Such indirect measurements are often difficult to determine and not reproducible as body circumferences are most often manually measured. Additionally, indirectly predicting body composition through complex anthropometric algorithms is time-consuming and inaccurate.

### 2.2. Bioelectrical Impedance Analysis

BIA offers a non-invasive method to estimate body composition by measuring electrical currents through the body to determine impedance. Using impedance data and prediction equations, BIA can estimate fat-free mass (FFM) and other body composition measures [22]. One limitation of BIA-generated estimates is that the analysis requires several assumptions. Still, the precision of modern BIA devices has been improved by incorporating multi-frequency currents and demographic data such as age, gender, and ethnicity [21].

Given its widespread use and improved accuracy, BIA is utilized by several digital biometry technologies as a means of measuring body fat and determining body composition. BIA has been incorporated into intelligent scales, handheld devices, and smartwatches. Smart scales developed by FitBit, Eufy, Garmin, and Withings employ BIA technology to provide individuals with estimates of their body composition from home. While accessible, easy to use, and low-cost, these devices have several disadvantages. They cannot measure visceral fat, muscle, and bone mass to provide an indirect estimation of fat mass. Furthermore, they are easily influenced by hydration status, food intake, and skin temperature [21]. Thus, although BIA-based technology can provide helpful insights into body composition, it is essential to recognize the limitations of using this technology in isolation to evaluate metabolic health.

## 3. Gold-Standard Measurement of Body Composition

The gold-standard assessment of body composition involves tomographic imaging techniques, including dual-energy X-ray absorptiometry (DEXA), computed tomography scan (CT scan), and magnetic resonance imaging (MRI) [23,24]. MRI and CT scans offer an accurate assessment of VAT, enabling its differentiation from SAAT. DEXA provides an accurate assessment of total fat mass and muscle mass [19,25,26,27,28,29]. However, these techniques are expensive, not routinely available, and can expose individuals to radiation or be contraindicated for patients with implanted devices. Therefore, these diagnostic tools are often limited to the research setting, as their high cost and limited accessibility create barriers to use in the clinical setting.

In sum, while the aforementioned techniques have their own benefits, they also each have unique limitations. These include the lack of reproducibility of body circumference measurements, the indirect prediction of body composition using complex anthropometric algorithms, high cost, limited availability, and exposure to radiation in some gold-standard biomedical imaging techniques. To overcome these limitations, newer techniques in digital biometry have emerged as potential tools to evaluate anthropometric and body composition measurements. Recent iterations of 2D and 3D optical image technologies might offer an innovative way to assess body circumferences and body composition that avoids some of these limitations.

## 4. Objective

The present work aims to critically review these digital biometric tools’ precision, efficacy, and limitations in providing accurate anthropometric measurements and body composition estimates and the adequacy of their application in the obesity field.

## 5. Methods

A systematic review was conducted using PubMed, Google Scholar, Public Library of Science (PLOS), Web of Science, and ScienceDirect to identify studies validating the use of 2D and 3D body scanners. A search strategy was utilized to identify published, full-text articles. Terms such as “2D body scanners”, “3D body scanners”, “anthropometry”, “waist circumference”, “hip circumference”, “body composition”, and “DEXA” were utilized in the search. Supplemental studies were also identified during the review of the collected articles and their references. The abstracts and studies identified were reviewed independently by three authors. The evaluation criteria for the automated body scan devices’ abilities to assess body circumferences and body composition were defined according to the AXIS-adapted critical appraisal tool, which evaluates the quality of cross-sectional studies [30]. Studies were excluded if they were not full-text or lacked sufficient reporting of the methods or results.

## 6. Results

### 6.1. 2D Body Scanners

Two-dimensional body scanning technology is emerging in digital biometry as a valuable and practical tool for assessing body composition. This technology uses unique processing algorithms to estimate anthropometric measurements, such as waist circumference, from 2D images obtained from cameras and smartphones. In contrast to traditional measurement methods, this technology is accessible, and in some cases, the data can be obtained without specialized training or equipment. This easy-to-use and readily available technology allows patients to obtain and monitor anthropometric measures at home, providing helpful information on their metabolic health. Several 2D body scanners are being developed to accurately analyze body measurements comparable to conventional anthropometry, including tape measures and calipers. These devices and the studies describing their use and efficacy are summarized in Table 1.

Several studies found that 2D optical imaging devices and programs generated body measurements that were similar to those obtained using manual anthropometric measurements. The Online Trial Room is a web-based application that calculates measurements of various body parts from 2D images of its users to provide them with clothing size estimates. Although tested in a small sample size, this application showed promise in generating accurate measurements and clothing size estimates. Compared to anthropometric measures, their device had root mean square error (RMSE) values of 0.808 for the neck, 1.478 for the shoulder, 4.454 for the upper waist, 3.83 for the lower waist, and 0.907 for the length, suggesting that this 2D optical imaging application can provide estimates of body measurements virtually without the need for in-person manual measurements [31]. The SmartFit Measurement2 application, which aims to give measurements of waist, lower hip, and thigh circumferences from front and side photos taken with a smartphone camera, similarly showed an average accuracy level of 95.59% with a small margin of error (0.5346 inches) when compared to body measurements obtained manually [32]. In a study of 41 individuals, Widyanti and colleagues used 2D imaging to provide estimates of various body circumferences and concluded that their estimates were not significantly different from those obtained by traditional methods, with comparable technical error of measurement (TEM) and reliability coefficients (r = 0.91–0.99) [35]. 

Others have shown that convolutional neural networks (CNNs) can be used to predict body measurements and body composition using 2D images taken via a smartphone. Souza and colleagues found that measurements generated by their 2D imaging processing application using the Dense Human Pose Estimation and Expectation-Maximization (EM) method provided accurate estimates of body measurements. In comparison with the skinfold measurements performed by a specialist, the mean squared error (MSE) was 0.0744 ± 0.0363 for the fist, 1.037 ± 0.863 for the forearm, 3.971 ± 2.309 for the breastplate, 1.836 ± 1.231 for the waist, 3.381 ± 4.704 for the relaxed biceps, 3.744 ± 3.734 for the thigh, and 4.606 ± 3.412 for the calf [33].

One study by Park and colleagues found that 2D body scanners can be used to provide estimates of weight in pediatric patients. In a study of 480 pediatric patients, they found that the Weighing Cam, an Android-compatible 2D body scanner, was able to accurately estimate pediatric body weight with a mean percent error (MPE) of 0.99%, mean absolute percentage error (MAPE) of 5.06%, and root mean square prediction error (RMSPE) of 11.32%. In comparison to the Broselow tape, today’s gold standard of estimating weight in pediatric emergency medicine, the Weighing Cam produced more precise estimates and showed smaller bias (mean difference = 1.98% (95% confidence interval: 2.91% to 1.05%) for MPE and 1.76% (95% confidence interval: 2.45% to 1.06%) for MAPE). The weight estimates produced by the Weighing Cam technology were more likely to be within 10% and 20% of the study subject’s actual weight (69.2% and 92.5%, respectively) than the Broselow tape (58.9% and 88.7%, respectively). The Weighing Cam could also produce weight estimates for all participants regardless of size, unlike the Broselow tape, which further exemplifies the potential of 2D technology to replace current practices of evaluating body size and measurements [34].

Beyond anthropometric measurements, 2D optical imaging has been shown to estimate body composition with comparable efficacy to advanced imaging techniques such as DEXA. In a study by Majmudar and colleagues, a novel computer vision method that utilizes CNNs, entitled Visual Body Composition (VBC), produced estimates of the percentage of total body fat from 2D images that were comparable to those generated by DEXA (Lin’s concordance correlation coefficient of 0.96). When compared to BIA methods, VBC had the lowest mean absolute error and standard deviation (2.16 ± 1.54%) from DEXA as well (*p* < 0.05 for all comparisons) [36]. Overall, this study showed that body composition estimates generated from 2D body scanning technology can be accurate and without bias when compared to the DEXA gold standard.

Overall, it is clear that 2D optical imaging techniques can provide reliable and accurate body measurements comparable to traditional anthropometric measures. Furthermore, these devices have several advantages over conventional measurements, including efficiency, convenience of home use, and affordability, which enhance their potential for widespread use. However, it is important to note that many of the studies reviewed were completed with small sample sizes, making these results difficult to extrapolate to the larger public. For instance, Anisuzzman and colleagues studied the Online Trial Room in 20 adults and failed to include details on the subjects’ ages, weights, or heights [31]. Similarly, Foysal and colleagues had a sample size of 12 individuals (10 men and 2 women) [32]. As a result, further work is needed to ensure that these technologies can provide reliable and accurate data in a diverse, large-scale population before their use is implemented in the clinical setting.

### 6.2. 3D Body Scanners

Similar to 2D optical imaging techniques, 3D scanners are able to rapidly and effectively generate detailed and precise anthropometric measurements. Several studies have demonstrated that 3D body scanners can accurately and reliably measure waist and hip circumferences similar to traditional anthropometric methods such as tape measures [37,38,39,40,41,42]. Furthermore, many of these devices have been validated in patients who have overweight or obesity [37,41,42]. The characteristics of these devices and the results of their validation studies are summarized in Table 2.

Beyond body circumference data, these advanced technologies utilize 3D imaging to construct models allowing for assessment of body composition such as fat mass, bone mass, and visceral fat. This technology’s detailed and comprehensive output is comparable to other whole-body imaging technology, including CT, MRI, and DEXA, without the cost and radiation exposure associated with these tools [44]. The validity and accuracy of 3D body scanners have been demonstrated in several studies. Bourgeois et al. found that the trunk volume estimates generated by the Styku S100 scanner correlated strongly with DEXA trunk volume estimates (R^2^ 0.98, *p* < 0.0001). Though the mean total body volume estimates differed from those generated by ADP, they also correlated strongly. In a study of 188 diverse patients, Bennett et al. found that the overall fat-free mass (FFM) calculated by the Styku S100 scanner showed a small mean difference of 1.2 ± 3.4 kg from that measured by DEXA. For fat mass (FM), the mean difference was 1.3 ± 3.4 kg. The concordance correlation coefficients (CCCs) for FFM and FM were 0.97 (95% CI: 0.96–0.98) and 0.95 (95% CI: 0.94–0.97), respectively, demonstrating the high agreement between the two modalities. The study also found no significant differences in the measurements when the groups were stratified by BMI, sex, and race/ethnicity [45]. Other studies have demonstrated that anthropometric measurements generated from these devices can be entered into complex prediction formulas to provide estimates of body fat percentage that are accurate and comparable to those of DEXA scans. For example, using the Size Stream SS20 3D optical system in combination with a 4C model, Harty and colleagues were able to predict body fat percentage with an R^2^ value of 0.78 compared to DEXA [46]. Similarly, Ng and colleagues were also able to accurately predict body composition using statistical modeling and the Fit 3D body scanner. Their estimates of fat mass and visceral fat were similar to the results from DEXA (fat mass R^2^ value: 0.88 in males and 0.93 in females; visceral fat R^2^ value: 0.67 in males and 0.75 in females) [47].

Three-dimensional body scanning has also been used to track changes in body shape by generating 3D avatars of the users. The technology was utilized for this purpose in a study including post-bariatric surgery patients by Kroh et al. In their study, they found that 3D body scanning was an accurate and effective means of tracking changes in body shape compared to traditional anthropometric data [48]. They also importantly showed that 3D body scanning was an effective means of measuring body composition over time in patients with obesity.

## 7. Discussion

This comprehensive review delves into the current landscape of digital biometry and available technological advancements, from smartphone applications to advanced imaging techniques, including 2D body scanners and 3D body scanners. Overall, the studies outlined demonstrate that these tools can provide accurate, precise, and reliable estimates of anthropometric measurements and body composition in individuals.

Both 2D and 3D body scanners provide body circumference estimates that correlate strongly with conventional anthropometry measurement techniques such as manual tape measurements. Three-dimensional scanners provide more precise and reliable measurements compared to 2D scanners, as 3D scanners provide a level of accuracy and detail that cannot be obtained from 2D scanners. In the majority of studies, the 3D optical devices had ICC values > 0.98, demonstrating that they can provide consistent reliable measurements that are superior to manual measurements [37,38,39,40,43]. Furthermore, compared to the studies involving 2D scanners, the 3D scanners have been validated and studied in larger, more diverse populations. Current studies involving 2D imaging are smaller in scale and include fewer patients with obesity, limiting the generalizability of their findings.

Beyond their superiority in circumference measurements, 3D scanners can provide accurate insights into body composition. These scanners have been shown to produce accurate and reliable estimates of FFM and FM that are comparable to today’s gold standards, including ADP and DEXA [41,46,47], while only one 2D scanner, the Visual Body Composition (VBC), could generate more advanced body composition data comparable to DEXA and CT [36]. Overall, both 2D and 3D imaging techniques are more accurate than BIA devices, which rely on indirect complex anthropometric algorithms to generate information on body composition. By accurately assessing body composition, 3D body scanners can provide more insight into one’s metabolic health.

Relative to DEXA and CT, 3D scanners are more affordable and accessible, but overall, they remain with limited availability and often require detailed training and instruction. The cost of purchasing a commercially available 3D scanner ranges from USD 400 to 240,000 depending on the technology, and this excludes any maintenance or software management fees [49]. Given their cost and the need for training and instruction in order to operate, they are mostly utilized in public settings such as gyms, health clinics, etc. These constraints impose a barrier for patients requiring serial monitoring who are homebound or lack the resources to access these settings routinely. This is an area that is evolving, however, as a pilot study was recently published on the use of contactless mobile 3D body scanning to assess anthropometric parameters in athletes. This study demonstrated that this mobile digital technology has promising perspectives when performed by trained users with rigorous data and appropriate software [50].

Two-dimensional body scanners, on the other hand, are often operated through smartphone technology, making them highly accessible and increasing their reach to patients at home. This provides a huge advantage for health monitoring, especially in clinics with limited resources and as healthcare delivery becomes increasingly virtual. Although many 2D technologies are not commercially available at this time, the costs associated with these programs will almost certainly be lower than those of 3D scanners. The use of remote monitoring should allow for obesity-related care to reach more patients and reduce the healthcare costs associated with diagnosing and treating obesity.

With these advantages, the novel technologies described in this review are emerging as a viable alternative to traditional body composition measurements and will potentially transform how obesity is diagnosed and managed. The reports generated by these tools can provide fast and accurate assessments of body composition and, therefore, metabolic health, aiding healthcare providers in more effectively identifying patients at risk of obesity-related health complications. These technologies also facilitate better monitoring of the efficacy of obesity interventions by accurately tracking changes in body composition. This enables physicians to determine if the interventions utilized by patients generate positive changes in body composition, such as loss of visceral adiposity, associated with reducing obesity-related health complications. Furthermore, research has shown that patients report improved experiences when using 2D images to monitor weight loss, which can enhance motivation and adherence [51].

Despite these advancements, there are notable limitations in optical imaging technologies. As noted, many of the studies reviewed involved small sample sizes, limiting their applicability to the general population. More research is needed with larger, more diverse populations, especially including patients with obesity, to better evaluate the accuracy and reliability of these devices in this population. Current iterations of 3D scanners have been shown to be less accurate in patients with obesity, which is an area that must be improved upon before these technologies are used routinely in the healthcare setting [52]. There is hope that as the technology advances, the camera and detection systems will become increasingly accurate, which is a phenomenon that has already been demonstrated in a study by Tinsley et al. In their study, the authors showed that the precision error and t-mean-square coefficient of variation were halved from the first-generation prototype of the Fit 3D Proscanner to the second-generation prototype [40]. Furthermore, the machine learning algorithms that these programs use to generate body composition reports will become increasingly accurate as more data are collected.

Lastly, further research is also necessary to evaluate the efficacy of these devices in clinical settings and their effectiveness in assessing altered body composition phenotypes and predicting obesity-related complications. There are early studies which have started to show that body composition data obtained by 3D imaging technology correspond accurately with other metabolic health parameters [38], but as this technology is adopted in the clinical setting, it is critical that the technology is validated as an accurate predictor of body composition in relation to metabolic health. Body scanning technology has important potential for use in future studies for monitoring changes in body composition related to obesity treatments, e.g., anti-obesity medication and bariatric/metabolic surgery, which is of particular interest given the concern for muscle mass loss associated with these interventions. It may also have utility in better predicting metabolically unhealthy phenotypes in some patients who otherwise have a normal BMI and could benefit from medical interventions to improve their overall metabolic health.

## 8. Conclusions

In conclusion, digital biometry represents a transformative advancement with the potential to better predict metabolic health. As the limitations of BMI and weight as the sole measures of obesity and associated metabolic health disturbances become more evident, the need for comprehensive body composition data grows increasingly critical. Digital biometry offers innumerable benefits over traditional body composition measurement tools such as calipers, scales, and tape measures, notably due to its ease of use and improved accuracy. Moreover, digital biometry, primarily in the form of 3D body scanning, is close to providing analyses of body composition with a depth and accuracy comparable to those of established imaging techniques such as DEXA and CT, while bypassing the logistical and economic barriers commonly associated with these methods. Preliminary studies have already illuminated the precision and reliability of these novel technologies, mainly 2D and 3D scanners, but continued research is needed to refine and validate these technologies for clinical application.

## Figures and Tables

**Table 1:** Summary of studies validating 2D body scanner applications.

Reference	Sample No. (n)	Characteristics	Objective	Results	Conclusion
Anisuzzaman et al., 2019 [31]	20	Web-based application, called Online Trial Room, that measures body dimensions from 2D images for clothing size.	Compare automatic measurements generated by image processing techniques to anthropometry.	Root mean square error (RMSE): neck 0.808, shoulder 1.478, upper waist 4.454, lower waist 3.83, length 0.907	Accurate predictions in 12 of 20 volunteers, but unable to accurately assess upper waist and lower waist measurements.
Foysal et al., 2021 [32]	12	Android-based application, entitled SmartFit Measurement, that measures waist, low hip and thigh circumferences from 2D images for accurate pant sizes.	Validate the application’s capability to correctly measure waist, low hip, and thigh circumferences compared to gold standard anthropometry.	Error range a with 95% CI: −0.72−0.34 inches, margin of error of 0.5346 in. 95.59% accuracy in measurements	No significant difference between application and manual measurements.
Souza et al, 2020 [33]	38	Computer-based program that uses digital image processing, CNNs and machine learning for body measurements.	Compare 2D image measurements obtained using CNNs and machine learning to skinfold measurements performed by a specialist.	Mean squared error (MSE) always below 4.606 ± 3.412 cm when using the Dense Human Pose Estimation and Expectation-Maximization (EM) approach	Overall accurate measurements that were similar to those obtained by specialists.
Park et al, 2020 [34]	480	Mobile app, The Weighing Cam, that estimates pediatric weight from 2D images.	Validate the accuracy of the application’s pediatric weight estimates compared to that of the Broselow tape.	Mean percent error (MPE) 0.99%, mean absolute percentage error (MAPE) 5.06%, and root mean square percentage error (RMSE) 11.32%. Compared to Broselow tape, the Weighing Cam had higher proportion of estimated weights within 10% of actual weights compared to Broselow tape (69.2% vs. 58.9%).	Estimates from imaging program were more accurate and precise than the Broselow tape.
Widyanti et al, 2007 [35]	41	Computer-based software generates body circumference measurements from digital images.	Compare digital measurements to manual measurement of 13 body parts.	Minimal differences between digital measurements and manual measurements with comparable TEM and reliability co-efficient.	Digital measurements of body circumferences are a valid and reliable alternative to manual measurements.
Majmudar, et al, 2022 [36]	134	Computer-based program, called Visual Body Composition (VBC), that uses 2D photos to estimate percentage total body fat (%BF) using a novel algorithm and convolutional neural networks (CNNs).	Evaluate the accuracy of VBC’s %BF estimates against BIA devices and ADP, with DXA as reference.	Mean absolute error (MAE) 2.16% ± 1.54%, MAPE 6.4%. Lowest MAE compared to all other devices (*p* < 0.05). Good concordance with DXA (CCC 0.96).	Most accurate and least biased method for estimating %BF compared to other devices.

**Table 2:** Summary of studies validating 3D body scanners for body circumference measurements.

Reference	Sample No. (n)	Characteristics	Objective	Results	Conclusion
Pepper et al., 2010 [37]	70	Portable 3-dimensional laser imaging device, called the Xu scanner, that measures body circumferences.	Compare the reliability and validity of a 3-dimensional laser body scanner to traditional anthropometry measurements with a tape measure.	- Standard error of the mean (SEM) for waist (WC) and hip circumferences (HC): 0.13 ± 0.13 and –0.24 ± 0.19 with *p* > 0.05. - All intraclass correlation coefficients (ICC) for circumference measurements ≥0.99.	The 3D laser yielded similar results to gold standard anthropometry and showed consistent measurements with minimal variations.
Jaeschke et al., 2015 [38]	60	Laser-based 3D body scanner device, Vitus^smart^XXL, that creates a 3D image and calculates body measurements.	Evaluate the accuracy and reliability of waist and hip circumferences generated by the 3D body scanner compared to manual anthropometry.	- WC correlation coefficients (r): 0.91–0.97 for men and 0.94–0.96 for women. - HC r: 0.65–0.97 for men and 0.80–0.98 for women. - ICCs for WC and HC measurements were >0.98.	WC and HC generated by the 3D body scanner were higher than manual anthropometry, but strongly correlated with anthropometry and were highly reliable.
Medina-Inojosa et al, 2016 [39]	83	Automated non-invasive 3D optical scanner entitled 3D Body Volume Index (BVI) scanning system, that produces body images and generates a maximum of 400 body measurements.	Assess reproducibility and reliability of anthropometric measures generated by 3D body scanner compared to anthropometry.	- 3D scanner variability: WC 1.3 ± 0.9cm and HC 0.8 ± 0.1cm. - *p*-value for difference in means between manual and automated measurements <0.05. - ICCs for all measurements >0.95.	3D body scanner showed lower variability in circumference measurements compared to manual measurements and were highly reliable.
Tinsley et al, 2023 [40]	69	Second-generation at-home 3D body scanner by Prism Labs, Inc. (Los Angeles, CA, USA)	Evaluate the precision of a 3D body scanner.	- WC: precision error (PE) of 0.5cm and root-mean-square coefficient of variation (RMS-%CV) of 0.6%. - HC: PE of 0.4 cm and RMS-%CV 0.4%. - ICCs for both WC and HC > 0.99.	3D body scanner showed precise and consistent measurements of WC and HC.
Ng et al, 2016 [41]	39	Commercially available 3D body scanner device, called Fit3D Proscanner (Fit3D, Redwood City, CA, USA), that generates a 360-degree body image and reports body circumferences.	Compare the accuracy of body circumference measurements generated by 3D scanner to manual anthropometry.	- Coefficient of determination (R^2^): WC= 0.95 and HC= 0.92. - Statistically significant mean differences between 3D scanner and tape measure: WC= 1.75 cm (95% CI 0.58–2.91 cm) and HC= 3.17 (95% CI 1.93–4.41 cm)	WC and HC generated by 3D scanner were strongly associated with those obtained using tape measurements, but there were significant mean differences between measurements.
Derouchey et al, 2020 [43]	49	Portable single stationary camera on a rotating platform, called the Styku S100, that generates 3D images and determines body circumference and composition measurements.	Assess the accuracy and test–retest reliability of the 3D scanner in determining body circumferences, surface areas, and volumes of athletes.	- Low random error (mean standardized typical error: scan 1 vs. 4., 0.14, 95% CI of 0.10–0.17) - Low systemic error (mean standardized bias: scan 1 vs. 4, 0.04, 95% CI: 0.02–0.06). - Strong test-retest correlations (mean ICC 0.98)	Styku S100 is a reliable tool to measure body circumference, surface areas, and volumes of athletes.
